# Effects of Caffeine on Egg Quality and Performance of Laying Hens

**DOI:** 10.3389/fvets.2020.545359

**Published:** 2020-09-25

**Authors:** Mailson da Silva Teixeira, Marcela Viana Triginelli, Thaís de Ataíde Costa, Leonardo José Camargos Lara, Benito Soto-Blanco

**Affiliations:** ^1^Department of Animal Science, Veterinary College, Universidade Federal de Minas Gerais, Belo Horizonte, Brazil; ^2^Department of Veterinary Clinics and Surgery, Veterinary College, Universidade Federal de Minas Gerais, Belo Horizonte, Brazil

**Keywords:** caffeine, coffee husks, chicken, eggs, laying hens

## Abstract

This study's objective was to determine the effects of caffeine intake at various levels, incorporated in the layers' food, on performance and egg quality of hens. A total of 576 hens, aged 56 weeks, were used. The layers were fed rations containing 0 (control), 150, 300, or 450 ppm of caffeine for 12 weeks. During the experimental period, performance parameters (weight, feed consumption, and livability) and egg production and quality (weight, Haugh unit, percentages of yolk, albumen and eggshell, yolk color, eggshell thickness, and resistance, and calcium and phosphorus eggshell contents) were evaluated. The highest concentration of caffeine in the diet (450 ppm) promoted a significant increase in the mortality of hens (1.45% per week) compared to controls (0.23%). There was a reduction in feed consumption by hens, decreased egg production, and reduced eggshell thickness and percentage, with the increase of caffeine. The egg yolk percentage was increased, and the eggshell percentage was reduced in the groups treated with 300 and 450 ppm of caffeine. Furthermore, reduced eggshell thickness was found in all groups that received caffeine. However, it was found that 150 ppm of caffeine in the food did not cause significant changes in most egg production and quality parameters. In summary, caffeine consumption by laying hens increased mortality rate and promoted deleterious effects on chicken production and egg quality at concentrations of 300 and 450 ppm.

## Background

Caffeine (1,3,7-trimethylxanthine) is a purine alkaloid synthesized using xanthosine formed from purine nucleotides (1). This compound is a known antagonist of adenosine A1, A2A, and A2B receptors in the central nervous system and peripheral tissues (2). Caffeine is found in plants such as coffee (*Coffea* spp.), tea plant (*Camellia sinensis*), cola (*Cola nitida*), cocoa (*Theobroma cacao*), maté (*Ilex paraguariensis*), and guaraná (*Paullinia cupana*) (1). Thus, humans regularly consume caffeine through the food and beverages derived from these plants; and in this process, large amounts of by-products are generated. These by-products can be used in animal feed, such as coffee husks (3), cocoa bean shell (4, 5), and green tea powder (6, 7). Due to the energy levels required by laying hens, fiber sources are essential, and they are being increasingly studied to improve their welfare due to changes in the speed of passage of food in the gastrointestinal tract and consequent reduction in mortality from cannibalism and to reduce the cost of feed (3). Wheat bran is the most used fiber source, but it has achieved high prices in tropical countries, and alternative fiber sources have been used.

Coffee husks contain high levels of fiber, and the concentration of crude proteins ranges from 6.9 to 12% (3, 8, 9). Coffee husks have been used as a component of food for animal species, including laying hens (3), pigs (10), sheep (8), and calves (9). Cocoa bean shells were found to contain 6.78–13.63% of crude protein and 12.75–33.0% of crude fiber (4, 5), and have been used for feeding a wide variety of livestock species (11). The addition of green tea powder to hens' food aims to improve egg nutritional quality by increasing vitamin E levels and reducing cholesterol and crude fat (7). The amount of caffeine in the fresh coffee husk is about 10.0 mg/g (12), from 1.59 to 4.21 mg/g in cocoa bean shells (13), and about 25.9 mg/g in green tea powder (7).

The quality of eggs produced was negatively affected in laying hens fed with by-products of caffeine-containing plants, such as coffee husks (3), cocoa bean shell (4), and green tea powder (6, 7). The adverse effects observed in egg production may be promoted by caffeine. However, no studies in the literature evaluated the effects of pure caffeine on egg quality, the performance of laying hens, and the safety of caffeine consumption.

Thus, the aim of this study was to determine the effects of caffeine intake (incorporated into hen feed at various levels) on the egg production and performance of laying hens. Our hypothesis is that caffeine consumption by laying hens might interfere with egg production and quality in a dose-dependent way. The generated data is useful for determining the amount of caffeine-containing plant by-products that could be used as feed ingredients, without affecting the health and performance of laying hens.

## Materials and Methods

### Animals

The experiment was conducted at the Experimental Farm “Prof. Hélio Barbosa” of the Veterinary School, Federal University of Minas Gerais (UFMG), located in the municipality of Igarapé, MG, Brazil. The Animal Use Ethics Committee of UFMG (protocol # 28/2018) approved all procedures described in this study. The possibility of animal death without euthanasia was stated in the study protocol submitted to the animal ethics committee because the expected mortality rate of laying hens ranges from 0.8 to 1.5% per week (14).

A total of 576 Lohmann LSL® laying hens aged 56 weeks were used. The hens were housed in a conventional, non-climatized laying house, equipped with cages at a density of 333 cm^2^/hen, with six hens per cage. Animal partitions were isolated by a wood splitter, preventing hens from accessing the feed from another partition. All hens were included in the experiment on the same date. Feeding was performed daily, and egg collection occurred four times a day. The light program used was 14 h of light/day, with 12 h of natural light and 2 h of artificial light (1 h of artificial light at dawn and 1 h in the early evening).

### Experimental Design

The hens were distributed in 24 experimental plots for 12 experimental weeks, 1 week after dietary standardization. The hens selected for the experiment had weight uniformity. The experimental design was completely randomized of four treatments with three different amounts of caffeine (anhydrous caffeine, Sulfal, Belo Horizonte, MG, Brazil) per ton of feed: 0 (control), 150, 300, and 450 g/ton. The highest concentration was based in an earlier study that showed impaired egg production by laying hens fed coffee husks at 42.5 g/kg (3), that is expected to contain caffeine at ~10.0 mg/g (12), equal to 425 g of caffeine/ton of feed. Ingredients and nutritional composition of the basal diet are shown in Table 1. Hens received water and feed *ad libitum*.

**Table 1:** Ingredients and nutritional values of the basal diet.

**Ingredients**	**%**
Corn grain	62.000
Soybean bran (45% CP^a^)	20.402
Wheat bran	3.000
Calcitic limestone	9.820
Meat and bone meal (40% CP)	4.000
Salt	0.380
Vitamin and mineral supplement^b^	0.200
DL-methionine	0.140
L-Lysine	0.040
Nutritional values	Amount
AMEn^c^ (kcal/kg)	2677.9954
Crude protein (%)	16.3585
Available phosphorus (%)	0.3205
Calcium (%)	4.0458
Sodium (%)	0.1821
Digestible lysine (%)	0.7470
Digestible Met+Cys (%)	0.5879
Digestible methionine (%)	0.3681
Digestible threonine (%)	0.5403

a *CP—crude protein*.

b *Composition per kg of product: Vitamin A: 8,000,000 UI, Vitamin D3: 2,100,000 UI, Vitamin E: 7,000 mg, Vitamin K3: 2,000 mg, Vitamin B_1_: 1,000 mg, Vitamin B_2_: 3,000 mg, Vitamin B_6_: 700 mg, Vitamin B_12_: 6,000 mg, Folic acid: 100 mg, Biotin: 10 mg, Niacin: 20 g, Pantothenic acid: 2,000 mg, Manganese: 55,000 mg, Zinc: 40,000 mg, Iron: 50,000 mg, Cupper: 6,000 mg, Cobalt: 100 mg, Iodine: 1,000 mg, Selenium: 200 mg, Calcium: 10,000 mg*.

c *AMEn—Nitrogen-corrected apparent metabolizable energy*.

Bodyweight was determined on the first and last days of the experimental period, and all hens from each partition were weighed together. Egg production was recorded daily; egg production per housed hen, and the percentage of weekly and total laying were calculated. Feed intake was determined weekly. Feed conversion was obtained by the ratio of the total feed consumed by the hens of each partition to the total weight of eggs laid in the same period (kg feed/kg eggs). The animals were monitored daily, and the number of hens was recorded daily to determine the mortality rate. All the research staff was previously trained for adequate animal care and handling.

### Analysis of Eggs

Eggs were collected after experimental periods of 3, 7, and 12 weeks, when the hens were 59, 63, and 68 weeks old, respectively. All eggs produced on these days were identified and weighed. Four eggs per repetition were randomly separated for assessments of egg quality. Evaluated parameters were weight, Haugh unit (HU), percentages of yolk, albumen and eggshell, yolk color, eggshell thickness and resistance, and eggshell calcium and phosphorus content.

HU was determined using a HU measuring device (Ames model S-8400, Massachusetts, USA). The calculation of HU was based on albumen height (H) and egg weight (W), as follows: HU = 100 log10 (H – 1.7 W0.37 + 7.56) (15). Percentages of yolk, albumen, and eggshell were determined by the methods described by Wu et al. (16). The yolk color score was determined immediately after the egg was broken, comparing the color of the yolk to a color fan (DSM Yolk Color Fan, 2005—HMB 51548, Basel, Switzerland). The same evaluator performed all measurements of the yolk color score in the same location.

Eggshell thickness was measured using a digital micrometer (Ames, Massachusetts, USA), with an accuracy of 0.001 mm, in three distinct regions of the shell (apical, equatorial, and basal); results were obtained by the average of measures of the three regions. Eggshell resistance was measured through the compression eggshell fracture force test, using a texture analyzer (TA-XT2, Stable Micro Systems, Surrey, England), as described by Carvalho et al. (17). Calcium and phosphorus eggshell contents were pretreated following the method described by the Brazilian Compendium of Animal Nutrition (18).

### Statistical Analysis

For the performance evaluations, the experimental design was entirely randomized (DIC), consisting of four treatments and six partitions with 24 hens in each.

For egg quality analysis, the experimental design was completely randomized, consisting of four treatments and 24 eggs per treatment, each egg being considered a repetition. For analysis of shell resistance, different eggs from the previously mentioned analysis (new sample collected) were used, likewise four eggs per repetition at 24 eggs per treatment with each egg being considered a repetition.

The results are presented as mean ± SEM. The data were analyzed with the aid of the SAS program and subjected to analysis of variance (ANOVA) to verify the significant effects between the simple factors; subsequently, a regression test between the treatments was performed. Additionally, ANOVA followed by Tukey test, and Kruskal-Wallis test followed by Student-Newman-Keuls were performed. The level of statistical significance was set at p < 0.05.

## Results

The body weights on the last day of the experiment were significantly lower (*p* < 0.001) in groups fed 300 and 450 ppm of caffeine than in controls (Table 2). The body weight gain was lower (*p* < 0.001) in all groups that received caffeine than control group, but the feed consumption in the entire experimental period was impaired (*p* < 0.001) only in hens fed the largest dose of caffeine.

**Table 2:** Initial and final body weight (in kg), body weight gain (in kg), and feed consumption (in g/day) of laying hens fed rations containing different concentrations of caffeine for 12 weeks.

**Caffeine in diet**	**Initial body weight (kg)**	**Final body weight (kg)**	**Bodyweight gain (g)**	**Feed consumption (g/hen.day)**	**Consumed caffeine (mg/hen)**
0 (control)	1.59 ± 0.01	1.60 ± 0.01^a^	16.8 ± 6.99^a^	108.4 ± 0.96^a^	0
150 ppm	1.63 ± 0.02	1.58 ± 0.01^a^	−51.3 ± 10.7^b^	109.1 ± 0.85^a^	16.365
300 ppm	1.63 ± 0.02	1.54 ± 0.01^b^	−97.8 ± 14.5^c^	106.4 ± 1.05^a^	31.920
450 ppm	1.60 ± 0.02	1.49 ± 0.01^c^	−113.5 ± 10.5^c^	98.3 ± 1.26^b^	44.235
*p*	n.s.	<0.001	<0.001	<0.001	–

*Data are shown as mean ± SEM*.

*^a, b, c^Different letters in the same column show significant difference (p < 0.05, ANOVA followed by Tukey test)*.

*n.s., non-significant*.

*n = 24*.

The number of hens that died during the experimental period was 4, 7, 12, and 25 in groups fed 0 (control), 150, 300, and 450 ppm of caffeine, respectively. All these hens were found dead without showing any noticeable sign of disease or pain, and the exact cause of death could not be determined. The log-rank test showed a significant difference (*p* < 0.0001) in mortality between the control and 450 ppm groups. However, there was no significant difference between the control group and the 150 ppm (*p* = 0.527) or the 300 ppm (*p* = 0.065) groups.

Both egg production and number of eggs per housed hen (Table 3) were significantly (*p* < 0.05) lower values in hens that consumed the highest concentration of caffeine in the diet. Feed conversion was worse in hens fed 450 ppm of caffeine vs. the control group.

**Table 3:** Number and percentage of produced eggs per laying hen and feed conversion in eggs (in g/egg) of hens fed rations containing different concentrations of caffeine for 12 weeks.

**Caffeine in diet**	**Number of eggs/laying hen**	**Percentage of eggs/laying hen**	**Feed conversion (kg feed/kg eggs)**
0 (control)	75.2 ± 0.63^a^	90.3 ± 2.21^a^	1.82 ± 0.33^b^
150 ppm	73.3 ± 1.24^a, b^	91.3 ± 1.64^a^	1.88 ± 0.38^a, b^
300 ppm	68.8 ± 1.22^c^	88.2 ± 1.33^a^	1.90 ± 0.46^a, b^
450 ppm	60.6 ± 2.27^d^	80.7 ± 2.19^b^	2.01 ± 0.67^a^
*p*	<0.001	<0.01	<0.05

*Data are shown as mean ± SEM*.

*^a, b, c, d^Different letters in the same column show significant difference (p < 0.05, ANOVA followed by Tukey test)*.

*n = 24*.

The average egg weight (Table 4) was reduced (*p* < 0.05) only in the group that received the highest caffeine dosage at 12 weeks of the experimental period. The HU (Table 5) showed higher values in eggs from hens fed all concentrations of caffeine vs. eggs from the control group after 12 weeks.

**Table 4:** Weight of the eggs (in g) produced by laying hens fed rations containing different concentrations of caffeine for 12 weeks.

**Caffeine in diet**	**Experimental period**		
	**4 weeks**	**8 weeks**	**12 weeks**
0 (control)	66.2 ± 1.06	66.4 ± 1.14	64.3 ± 0.96^a^
150 ppm	65.0 ± 1.03	65.5 ± 0.92	64.1 ± 0.80^a, b^
300 ppm	64.1 ± 0.98	63.1 ± 0.82	64.1 ± 1.04^a^
450 ppm	64.2 ± 0.73	64.3 ± 1.09	60.5 ± 1.07^b^
*p*	n.s.	n.s.	<0.05

*Data are shown as mean ± SEM*.

*^a, b^Different letters in the same column show significant difference (p < 0.05, ANOVA followed by Tukey test)*.

*n.s., non-significant*.

*n = 24*.

**Table 5:** Haugh units of the eggs produced by laying hens fed rations containing different concentrations of caffeine for 12 weeks.

**Caffeine in diet**	**Experimental period**		
	**4 weeks**	**8 weeks**	**12 weeks**
0 (control)	91.8 ± 0.74^c^	87.9 ± 1.10	86.8 ± 1.02^b^
150 ppm	92.9 ± 0.78^b, c^	87.5 ± 1.05	92.9 ± 0.83^a^
300 ppm	94.6 ± 1.25^a, b^	90.1 ± 1.10	91.5 ± 0.93^a^
450 ppm	95.7 ± 0.89^a^	90.2 ± 1.03	94.5 ± 1.08^a^
*p*	0.01	n.s.	<0.001

*Data are shown as mean ± SEM*.

*^a, b, c^Different letters in the same column show significant difference (p < 0.05, Kruskal-Wallis test followed by Student-Newman-Keuls test)*.

*n.s., non-significant*.

*n = 24*.

The percentages of egg components (yolk, albumen, and eggshell) of the produced eggs are shown in Table 6. Consumption of 300 and 450 ppm of caffeine in the diet increased the egg yolk percentage after 12 weeks of the experiment. Also, caffeine intake was responsible for reducing the eggshell percentage at all evaluations. On the other hand, the albumen percentage was not affected.

**Table 6:** Percentages of yolk, albumen, and eggshell of the eggs produced by laying hens fed rations containing different concentrations of caffeine for 12 weeks.

**Caffeine in diet**	**Experimental period**		
	**4 weeks**	**8 weeks**	**12 weeks**
**Percentage of yolk**			
0 (control)	28.1 ± 0.42	27.9 ± 0.41	27.4 ± 0.27^a^
150 ppm	28.0 ± 0.40	28.6 ± 0.35	27.6 ± 0.41^a, b^
300 ppm	28.4 ± 0.37	28.9 ± 0.45	28.4 ± 0.57^b^
450 ppm	28.2 ± 0.40	28.3 ± 0.30	28.9 ± 0.49^b^
*p*	n.s.	n.s.	<0.05
**Percentage of albumen**			
0 (control)	62.3 ± 0.45	62.5 ± 0.47	62.7 ± 0.32
150 ppm	62.6 ± 0.43	62.3 ± 0.44	63.7 ± 0.38
300 ppm	62.6 ± 0.40	62.1 ± 0.44	63.0 ± 0.63
450 ppm	62.9 ± 0.42	63.0 ± 0.34	62.4 ± 0.50
*p*	n.s.	n.s.	n.s.
**Percentage of eggshell**			
0 (control)	9.61 ± 0.14^a^	9.64 ± 0.11^a^	9.87 ± 0.14^a^
150 ppm	9.42 ± 0.11^a^	9.16 ± 0.14^a, b^	8.72 ± 0.18^b^
300 ppm	9.04 ± 0.12^b^	8.95 ± 0.10^b^	8.56 ± 0.20^b^
450 ppm	8.93 ± 0.14^b^	8.73 ± 0.15^b^	8.78 ± 0.17^b^
*p*	<0.001	<0.001	<0.0001

*Data are shown as mean ± SEM*.

*^a, b^Different letters in the same column show significant difference (p < 0.05, Kruskal-Wallis test followed by Student-Newman-Keuls test)*.

*n.s., non-significant*.

*n = 24*.

Thickness and strength of the eggshell and the yolk color score are presented in Table 7. At all concentrations, caffeine intake was responsible for lower (*p* < 0.05) eggshell thickness at all evaluated periods. However, caffeine reduced (*p* < 0.05) the strength of the eggshell after 4 weeks of the experiment, but there was no significant difference at later periods. Furthermore, there was interference (*p* < 0.05) in the yolk color score of eggs from laying hens exposed to caffeine after 8 and 12 weeks of the experimental period.

**Table 7:** Thickness and strength of the eggshell and the yolk color score of the eggs produced by laying hens fed rations containing different concentrations of caffeine for 12 weeks.

**Caffeine in diet**	**Experimental period**		
	**4 weeks**	**8 weeks**	**12 weeks**
**Thickness of eggshell (in mm**^**2**^**)**			
0 (control)	39.4 ± 0.63^a^	39.6 ± 0.48^a^	39.6 ± 0.54^a^
150 ppm	38.0 ± 0.36^b^	37.5 ± 0.37^b^	35.6 ± 0.66^b^
300 ppm	36.58 ± 0.44^b^	36.5 ± 0.39^b^	35.0 ± 0.68^b^
450 ppm	36.5 ± 0.59^b^	36.1 ± 0.65^b^	35.6 ± 0.73^b^
*p*	<0.0001	<0.0001	<0.0001
**Strength of eggshell (in kg/cm**^**2**^**)**			
0 (control)	5.19 ± 0.17^a^	4.41 ± 0.16	4.88 ± 0.24
150 ppm	3.33 ± 0.16^b^	4.21 ± 0.21	4.64 ± 0.20
300 ppm	3.27 ± 0.11^b^	4.20 ± 0.17	4.21 ± 0.19
450 ppm	3.53 ± 0.15^b^	4.49 ± 0.19	4.35 ± 0.21
*p*	<0.0001	n.s.	n.s.
**Yolk color score**			
0 (control)	7.25 ± 0.14	6.37 ± 0.12^b^	6.00 ± 0.20^c^
150 ppm	7.08 ± 0.13	7.25 ± 0.12^a^	6.58 ± 0.16^b, c^
300 ppm	6.96 ± 0.09	6.96 ± 0.09^a^	7.04 ± 0.23^b^
450 ppm	7.04 ± 0.13	7.04 ± 0.13^a^	7.25 ± 0.23^a^
*p*	n.s.	<0.0001	<0.001

*Data are shown as mean ± SEM*.

*^a, b, c^Different letters in the same column show significant difference (p < 0.05, Kruskal-Wallis test followed by Student-Newman-Keuls test)*.

*n.s., non-significant*.

*n = 24*.

## Discussion

Feed consumption was impaired in hens from the 450 ppm caffeine group. Caffeine might interfere with appetite, reducing energy intake (19, 20). However, the mechanism underlying this effect is still unclear. Furthermore, ingesting caffeine increases corporal energy consumption. Caffeine was found to increase the serum release of catecholamines (epinephrine, norepinephrine, and dopamine) (21) and the oxidation of lipids (22, 23).

The group fed 450 ppm caffeine showed an increased mortality rate, with a mortality rate of 1.45% per week, whereas the mortality rates of hens fed 0, 150, and 300 ppm caffeine were 0.23, 0.41, and 0.69%, respectively. The acceptable mortality rate for caged laying hens is up to 1.2% per week (14), then the group fed 450 ppm caffeine was the only one showing a high mortality rate. Increased mortality rates were also observed in broiler chicks that received caffeine. These chicks developed pulmonary hypertension syndrome or ascites, with right ventricular hypertrophy and an increased hematocrit (24). Furthermore, the stimulating effect of caffeine may induce the observed reduced feed consumption, generating an energy and protein deficit. Also, caffeine interferes with the proper function of the immune system (25, 26), which may have increased the susceptibility of hen chickens to infections.

The quality of produced eggs was negatively affected by feeding laying hens with by-products of caffeine-containing plants, such as coffee husks (3), cocoa bean shell (4), and green tea powder (6, 7). In this study, caffeine ingestion negatively affected the production and quality of eggs. The egg production per hen was reduced in those fed rations containing 300 or 450 ppm of caffeine. The egg weight was also reduced in the groups that consumed the diet containing the highest amount of caffeine after 12 weeks. Additionally, after 12 weeks, the percentage of yolk and the yolk color score increased in groups treated with 300 and 450 ppm of caffeine, and the HU was augmented in all groups that received caffeine. The reduced egg weight and increased yolk percentage, yolk color score, and HU are probable consequences of the eggs' lower water content.

The interference of caffeine with egg production can be attributed to the observed reduction in feed consumption that could, in turn, generate an energy and protein deficit. Another hypothesis is that caffeine might interfere directly with ovarian physiology. In a previous study, female rats fed caffeine showed increased ovarian production of estradiol and delayed vaginal opening (27). Furthermore, several studies evaluating women showed the consumption of coffee and other caffeinated beverages was linked to a longer delay before becoming pregnant. This observed effect was proportional to the number of ingested beverages (28–30), but there is no consensus that caffeine promotes this effect (31).

Another effect of caffeine observed in the present study was the harmful interference with the eggshell, characterized by the reduced thickness and shell percentage at all evaluations. These effects were also observed in laying hens fed rations containing 4.25% of coffee husk (3). The reduced thickness and percentage of eggshell may be attributed to caffeine interference on calcium metabolism due to increased urinary excretion of calcium and magnesium (32, 33). Caffeine may promote hypocalcemia due to an increased compensatory bone resorption rate and reduce bone mineral density in laboratory animals (34). In humans, caffeine intake at high doses increases the risk of developing osteopenia and osteoporosis (35) and, consequently, augments the possibility of fractures (36). Thus, it is feasible to suppose that caffeine ingestion raised the urinary calcium excretion of laying hens, resulting in a smaller amount of this mineral available for eggshell formation.

## Conclusions

The present study reveals that caffeine consumption by laying hens increased the mortality rate and promoted deleterious effects on hen performance and egg quality when its concentration in the diet was 300 or 450 ppm. Consumption of up to 150 ppm of caffeine in the diet did not significantly interfere in hens or egg production. This concentration of caffeine in the diet is equivalent to ~15 kg of coffee husk, 35.6 kg of cocoa bean shell, and 5.7 kg of green tea powder per ton of feed, considering that the caffeine content in these compounds is ~10.0 mg/g (12), 4.21 mg/g (13), and 25.9 mg/g (7), respectively. However, doses above this value may have undesirable consequences on egg production and quality.

## Data Availability Statement

The raw data supporting the conclusions of this article will be made available by the authors, without undue reservation.

## Ethics Statement

This animal study was reviewed and approved by Animal Use Ethics Committee of the Universidade Federal de Minas Gerais—UFMG.

## Author Contributions

BS-B and LL conceived and designed the experiments. MST, MVT, and TC performed the experiments. BS-B, LL, and MST analyzed the data. BS-B and MST drafted the manuscript. All authors read and approved the final manuscript.

## Conflict of Interest

The authors declare that the research was conducted in the absence of any commercial or financial relationships that could be construed as a potential conflict of interest.
